# Comparative Analysis of Hyperbilirubinemia Between Congenital Hypothyroidism and Euthyroidism in Neonates in Rawalpindi, Pakistan

**DOI:** 10.7759/cureus.100944

**Published:** 2026-01-06

**Authors:** Mafia Arshad, Anam Hanif, Naila Yasin, Abdul Ali Khan, Ali Husnain, Hashmatullah Stanikzai, Atif Ayub, Hina Meraj, Alishba Amin, Ali Hamza

**Affiliations:** 1 Life Sciences, Afro-Asian Institute, Lahore, PAK; 2 Life Sciences, University of Sargodha, Sargodha, PAK; 3 Life Sciences, COMSATS (Commission on Science and Technology for Sustainable Development in the South) University, Islamabad, PAK; 4 Psychology, University of New Mexico, Albuquerque , USA; 5 Laboratory, Islamabad Diagnostic Center, Islamabad, PAK; 6 Life Sciences, Zohra institute of Health Sciences, Government College University, Faisalabad (GCUF), Rawalpindi, PAK; 7 Medicine, Shaikh Khalifa bin Zayed Al Nahyan Medical and Dental College, Lahore, PAK; 8 Life Sciences, Center of Advanced Studies in Health and Technology, Rawalpindi, PAK; 9 General Practice, Seha Salma Rehabilitation Hospital, Abu Dhabi, ARE; 10 Life Sciences, Pakistan Institute of Medical Sciences, Islamabad, PAK

**Keywords:** congenital hypothyroidism (ch), hyperbilirubinemia, newborn jaundice, thyroid stimulating hormone tsh, total serum bilirubin (tsbr)

## Abstract

Introduction: Neonatal hyperbilirubinemia is a prevalent clinical observation that, if persistent or severe, may suggest underlying endocrine abnormalities such as congenital hypothyroidism (CH). Thyroid hormones play a crucial role in liver function and the metabolism of bilirubin.

Objective: This study aimed to evaluate blood bilirubin and thyroid stimulating hormone (TSH) levels in neonates diagnosed with CH compared to those with normal thyroid function (euthyroid).

Material and methods: A comparative cross-sectional observational study was conducted at Majeed Medical & Gynae Complex and Farid Medical Family Hospital in Rawalpindi, Pakistan. A total of 200 neonates, aged 1-30 days, were included in the study, comprising 100 confirmed cases of CH and 100 controls. The clinical features and demographics of patients were recorded using a structured interview questionnaire. Serum bilirubin and TSH levels were measured using automated Roche analyzers (C-311 and E-411). Data were analyzed utilizing IBM SPSS Statistics for Windows, version 25 (IBM Corp., Armonk, New York, United States) and GraphPad Prism (Dotmatics, Boston, Massachusetts, United States).

Results: Prolonged jaundice was the most prevalent (74%) clinical finding among the CH group. Mean bilirubin (total, direct, and indirect) levels and TSH levels were analyzed using an independent t-test, with a significance threshold set at p < 0.01. A positive significant correlation exists between TSH levels and bilirubin (total and indirect) concentration in the hypothyroid group, with a p-value of < 0.01. These findings highlight the clinical significance of timely newborn TSH screening and the early administration of levothyroxine to mitigate jaundice-related morbidity and prevent unnecessary phototherapy or extended hospitalization.

Conclusion: The current study concludes that newborns with CH have markedly increased all types of bilirubin levels relative to euthyroid newborns, and there is a statistically significant positive correlation between TSH levels and bilirubin levels (total and indirect).

## Introduction

Congenital hypothyroidism (CH) is a thyroid hormone deficiency present at birth, resulting from abnormal thyroid gland development, hormone synthesis defect, or central hypothalamic pituitary dysfunction [1], with one in 3000-4000 live births globally, with higher rates in Hispanic (one in1600) and Asian (one in 2380) populations [2].As of 2025, only 30% of newborns worldwide undergo CH screening, with significant gaps in Africa and parts of Asia [3]. In Pakistan, CH occurs at a rate of one in 1100-1500 live births, significantly higher than the global average [4]

Causes of primary CH (80-90% of cases) include thyroid dysgenesis (agenesis, ectopic thyroid, or hypoplasia) [2] and genetic mutations affecting enzymes DUOX2 or TG [5], which result in dysfunctions of thyroid hormone production. The incidence of central CH falls within 1:13,000-30,000 cases because of pituitary/hypothalamic dysfunction, which has genetic roots connected to *TSHB*, along with IGSF1 or multiple pituitary hormone deficiencies (MPHD) [6]. Transient CH occurs due to maternal antibodies (e.g., thyroid-blocking immunoglobulins), iodine excess/ deficiency, or anti-thyroid drugs during pregnancy [2].

CH is primarily diagnosed through newborn screening, which measures TSH and T4 levels in heel-prick blood spots, with confirmatory venous testing for abnormal results to assess free T4 and TSH levels. Imaging techniques like thyroid ultrasound or scintigraphy are used to identify structural abnormalities (e.g., ectopic or absent gland) [2]. Scientific detection of central CH needs T4-based assessment methods instead of TSH-only screening because it might miss the condition [7]. The essential treatment process includes starting levothyroxine (L-T4) at 10-15 µg/kg/day before the two-week mark to restore thyroid function while stopping developmental problems. [2] Clinical jaundice develops in 60-80% of normal-term and late-preterm babies, yet low-and middle-income countries report higher rates due to screening and treatment restrictions [8]. Neonatal hyperbilirubinemia develops from increased bilirubin production (hemolysis) and inadequate conjugation because of immature UGT1A1 activity, alongside illnesses and delayed feeding status as additional risk elements [9]^. ^Two leading groups of factors now dominate available evidence regarding neonatal hyperbilirubinemia. The primary causes of this condition are G6PD deficiency [10]and genetic polymorphism (e.g., Gilbert syndrome) [11], which produce indirect bilirubin levels exceeding 80% of total serum bilirubin (TSB).

The condition of conjugated hyperbilirubinemia (direct bilirubin > 20% of TSB) normally indicates hepatobiliary problems, including biliary atresia or sepsis [12]. A TSB amount greater than 20 mg/dl among infants older than 35 weeks of gestation creates substantial acute bilirubin encephalopathy (ABE) and kernicterus dangers when coupled with hemolytic diseases, sepsis, or hypoalbuminemia. The medical urgency of ABE appears as lethargy combined with hypertonia and high-pitched crying that progresses through seizures and coma if untreated. Kernicterus develops as the permanent neurological consequences of bilirubin damage, causing dystonia along with hearing deficits and vision problems [13]. Therefore, scientists need to study the endocrine system that affects bilirubin metabolism since this pathway appears important. New research shows a vital relationship exists between jaundice in newborns and thyroid function disorders, specifically hypothyroidism, because this condition impairs hepatic conjugation through UDP-glucuronosyltransferase (UGT1A1) activity [14]. An Iranian epidemiological study showed hypothyroidism to be responsible for 4.2% of all cases of hyperbilirubinemia [15].Additionally, next-generation sequencing revealed compound heterozygous mutations in the *DUOX2* gene, consisting of c.244 (exon 4) C>T and c.1883 (exon 16) deIA. Scientists obtained control of the thyroxine synthesis-regulating enzyme known as peroxidase.

Few studies exist that systematically examine bilirubin levels between hypothyroid and euthyroid neonates, although CH shows a genetic connection to *DUOX2* mutations in 21-45% of cases [16,17]. Even though several international studies indicate that CH is associated with neonatal hyperbilirubinemia [18,19], the majority report epistemological data for (such as *DUOX2*-related) or genomic-plus (planned genome-wide association study (GWAS)) studies of total, direct, and indirect serum bilirubin between hypothyroid and euthyroid neonates have not conducted a direct and systematic comparison yet [16,17]. In addition, there is a paucity of information regarding this subject in South Asian populations, and to the best of our knowledge, no published study from Pakistan looked specifically at bilirubin levels in neonates with CH as compared with euthyroid controls.

For a setting where screening for newborn thyroid has not yet been universally introduced, this represents a significant regional and clinical gap. The comparison of such a difference in structure has not been done to the best of our knowledge; therefore, the present study was designed to bridge this gap by generating the first comparative data from Rawalpindi, Pakistan, with respect to bilirubin fractions and their relationship with TSH in congenital hypothyroid versus euthyroid spectrum in neonates, which can help shape early screening and management plans. This study aims to investigate the role of thyroid dysfunction in determining the severity and duration of neonatal hyperbilirubinemia

## Materials and methods

This was a comparative observational cross-sectional study conducted at Majeed Medical & Gynae Complex and Farid Medical Family Hospital, Rawalpindi, Pakistan, from May 2024 to September 2024. The study was approved by the Institutional Research Board of Zohra Institute of Health Sciences (reference number: ZIHS/IRB/2024/1026).

Eligibility criteria

Inclusion Criteria

This study involved neonates who fulfilled defined eligibility criteria. Neonates diagnosed with CH constituted the case group, whereas those with normal thyroid function were designated as the control group. Both groups included neonates ranging from one to 30 days of age. Inclusion criteria were limited to individuals who had not initiated thyroid-replacement therapy before the collection of primary laboratory data, or whose therapy initiation schedules were explicitly documented, to ensure precise interpretation of thyroid function and bilirubin parameters.

Exclusion Criteria

Neonates with pre-existing neonatal diabetes were excluded from the study. Infants with diagnoses of hemolytic disease, sepsis, notable congenital anomalies, hepatic or biliary disorders, or a history of exchange transfusion were excluded from the study. Neonates who received prolonged phototherapy before baseline assessment or had incomplete medical records were excluded from the analysis. Neonates born before 34 weeks of gestation, classified as extremely preterm, were excluded to reduce potential confounding variables. Additionally, instances where written informed consent from parents or guardians was not acquired were excluded from the study.

Sample Size Calculation

A formal EPI tool was used to select the sample size based on the prevalence of CH in the selected study region. Due to the absence of prior regional data on bilirubin levels in neonates with CH, a formal a priori power calculation was not conducted. The sample size was determined based on the disease prevalence in the study region and the number of eligible neonates presenting during the study period. The main aim was to discern initial differences and trends among groups and to formulate hypotheses for subsequent, sufficiently powered studies.

**Figure 1:**
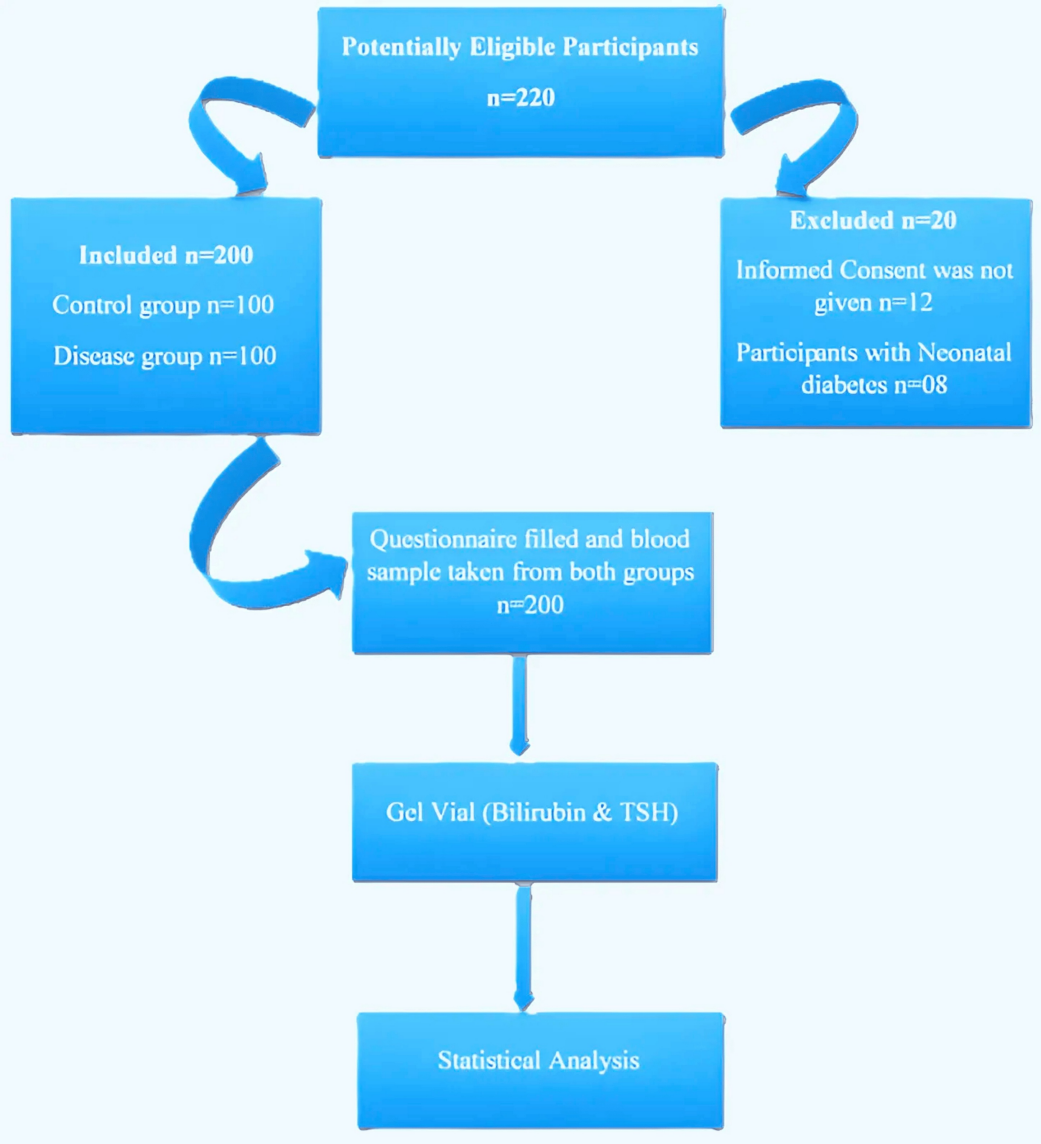
Schematic diagram of participant selection and study process TSH: thyroid-stimulating hormone

Participants were recruited through convenience sampling, contingent upon the availability of eligible neonates throughout the study period. Controls were chosen from neonates visiting the same facilities and were matched based on age, sex, and ethnicity (Pakistani). A total of 200 neonates, 100 with CH (disease group) and 100 healthy neonates (control group) of the same age and sex, were included in the study (Figure 1). No randomization was performed due to the study's cross-sectional nature.

Control group selection: The control group selection was done through a questionnaire (see Appendices), and there was no indication of a family or personal history of hypothyroidism or any other illness in a single healthy participant. The ethnicity of the patients and controls (Pakistani) was ascertained using an oral questionnaire. TSH levels were measured to check the normal thyroid function of the control group.

Sample collection and lab analysis

Written informed consent was obtained from all patients and controls before the collection of samples for laboratory investigations. The clinical features and demographics of patients were recorded using a structured interview questionnaire (see Appendices). A blood sample was taken in a gel vial for bilirubin and TSH analysis. After that, samples were centrifuged to separate the serum. Then, from the serum, bilirubin levels and TSH levels were measured using automated Roche analyzers (C-311 and E-411; Roche Holding AG, Basel, Switzerland).

Statistical analysis

Before conducting inferential analysis, equality of variances was confirmed using an F-test and by visually inspecting histograms. Given that bilirubin and TSH values approximated a normal distribution, parametric tests were deemed suitable. Between-group comparisons were conducted using the unpaired Student’s t-test, while correlations were assessed with Pearson’s correlation coefficient. GraphPad Prism (Dotmatics, Boston, Massachusetts, United States) was used for visual representation of results (e.g., bar graphs, scatter plots), while IBM SPSS Statistics for Windows, version 29 (Released 2022; IBM Corp., Armonk, New York, United States) was used for statistical comparisons and descriptive statistics. 

## Results

This investigation examined a total of 200 patients, all of whom were of Pakistani ancestry. The neonates in the trial were categorized into two groups based on thyroid function: control (normal) and disease (hypothyroidism) groups. Both groups were divided into five categories based on their age. The predominant number of patients in the control group (n=40, 40%) and the disease group (n=38, 38%) were within the 1-6 days age category, followed by the 7-12 days age category (n=27, 27% and n=28, 28%, respectively). The gender distribution in both groups was as follows: 52 (52%) male and 48 (48%) female neonates in the normal group, and 53 (53%) male and 47 (47%) female neonates in the diseased group (Table 1). In the study, 95 patients (95%) with CH had no familial history. According to their clinical findings, persistent jaundice was the predominant observation (n=74, 74%) (Table 2).

**Table 1:** Age and gender of neonates in the two groups

Variable	Category	Control Group		Disease Group	
		Frequency	Percentage	Frequency	Percentage
Age (Days)	1–6	40	40%	38	38%
	7–12	27	27%	28	28%
	13–18	13	13%	11	11%
	19–24	10	10%	12	12%
	25–30	10	10%	11	11%
Gender	Male	52	52%	53	53%
	Female	48	48%	47	47%

**Table 2:** Family history and clinical findings in the neonates with congenital hypothyroidism (disease group) (N=100)

Variable	Category	Frequency	Percentage
Family History	Yes	5	5%
	No	95	95%
Clinical Findings	Normal	3	3%
	Prolonged-Jaundice	74	74%
	Dry-Skin	3	3%
	Constipation	16	16%
	Microcephaly	4	4%

**Figure 2:**
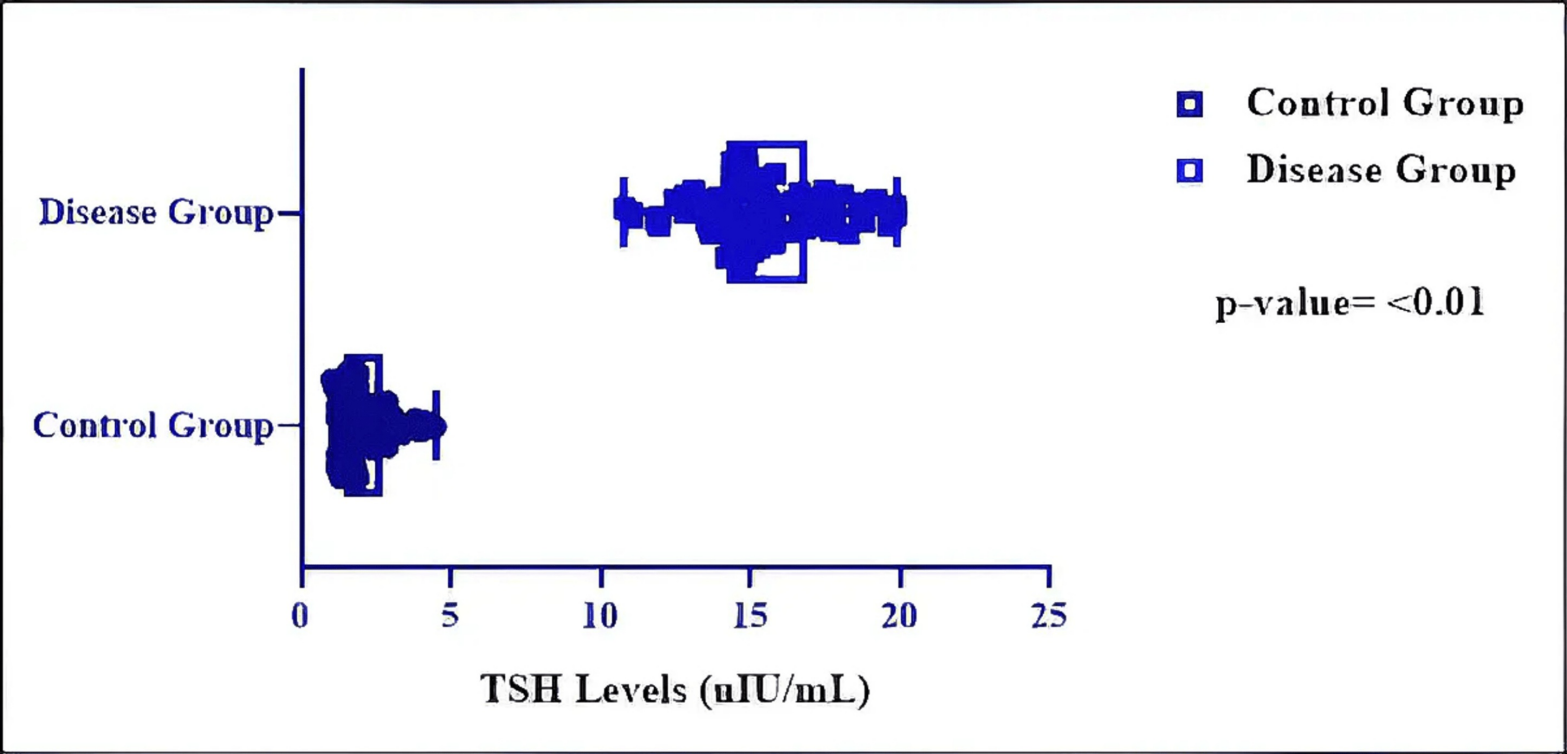
Comparative analysis of TSH levels between neonates in the two groups (unpaired t-test) TSH: thyroid stimulating hormone

The study demonstrated a statistically significant disparity between the control group and the illness group across all assessed parameters (p < 0.01) (Table 3). Serum TSH concentrations were significantly elevated in infants with CH relative to the control group (mean 15.43 vs. 2.13 µIU/mL; mean difference = 13.30 µIU/mL; 95%CI: 12.85-13.74; p < 0.01, unpaired two-tailed t-test). The size of this difference was substantial, with an eta-squared (η²) value of 0.946, signifying that roughly 95% of the variance in TSH levels was due to thyroid function. Although the F-test indicated unequal variances between groups (p < 0.0001), the use of an unpaired t-test was retained due to equal group sizes and the robustness of the test under these conditions (Figure 2).

**Table 3:** Descriptive and unpaired t-test analysis between control and disease group TSH: thyroid-stimulating hormone

Variable	Control Group (n=100), mean±SD	Disease Group (n=100), mean±SD	t-value	p-value
TSH (uIU/mL)	2.13 ± 0.86	15.43 ± 2.09	58.96	< 0.01
Total bilirubin (mg/dL)	5.92 ± 2.71	15.60± 2.38	26.81	< 0.01
Direct bilirubin (mg/dL)	2.48 ± 1.44	4.27 ± 2.03	7.18	< 0.01
Indirect bilirubin (mg/dL)	3.52 ± 2.74	11.42 ± 3.23	18.67	< 0.01

**Figure 3:**
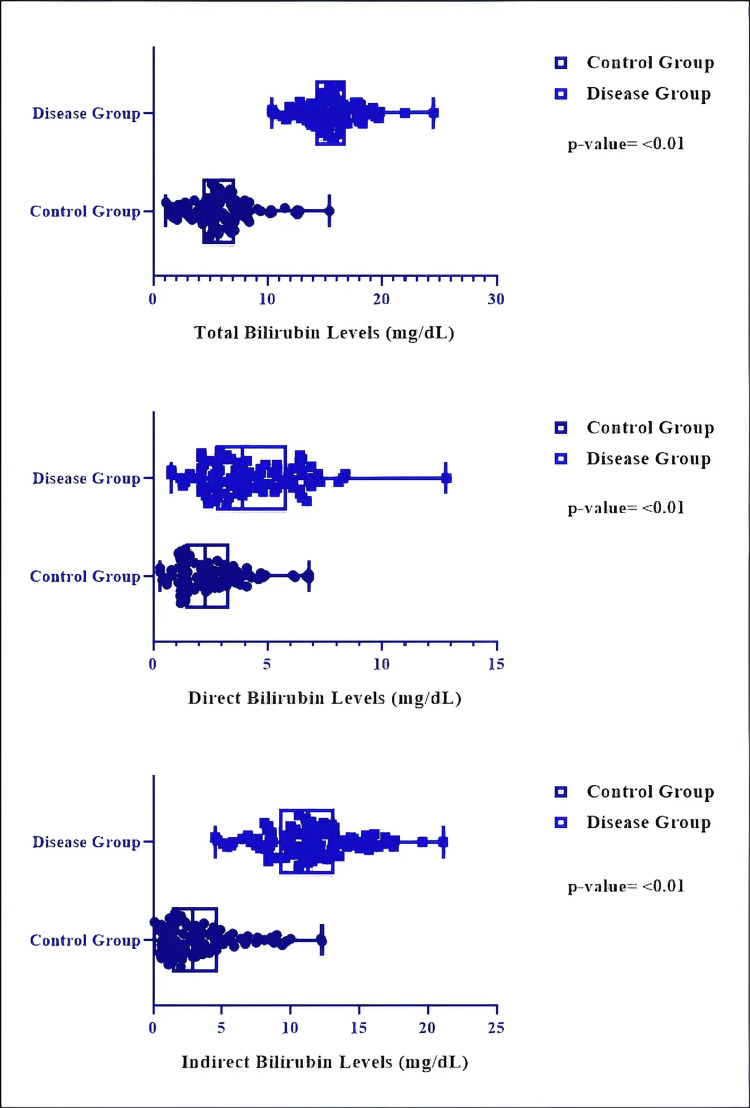
Comparative analysis of bilirubin levels between the two groups (unpaired t-test)

Neonates with CH had significantly elevated total blood bilirubin levels compared to the control group (15.60 ± 2.38 vs. 5.92 ± 2.71 mg/dL; mean difference = 9.67 mg/dL; 95% CI: 8.96-10.39; p < 0.01, unpaired two-tailed t-test). The difference was substantial, with an eta-squared (η²) value of 0.784, signifying that around 78% of the variance in total bilirubin levels was due to thyroid status. Equality of variances was confirmed using an F-test (p = 0.19), supporting the appropriateness of parametric analysis (Figure 3).

Direct bilirubin concentrations were markedly elevated in infants with CH relative to the control group (mean 4.27 vs. 2.48 mg/dL; mean difference = 1.79 mg/dL; 95%CI: 1.30-2.28; p < 0.01, unpaired two-tailed t-test). The difference was moderate, with an eta-squared (η²) value of 0.206, signifying that almost 21% of the variance in direct bilirubin levels was due to thyroid status. An F-test indicated uneven variances among groups (p = 0.0007); yet, due to the equal group sizes and the t-test's robustness under these circumstances, parametric analysis was maintained (Figure 3).

Neonates with CH had significantly elevated indirect bilirubin levels compared to the control group (mean 11.42 vs. 3.52 mg/dL; mean difference = 7.90 mg/dL; 95%CI: 7.07-8.74; p < 0.01, unpaired two-tailed t-test). The size of this difference was substantial, with an eta-squared (η²) value of 0.638, signifying that about 64% of the variance in indirect bilirubin levels was ascribed to thyroid status. Homogeneity of variances was supported by the F-test (p = 0.10), further justifying the use of parametric testing.(Figure 3) The results demonstrate a notable distinction between CH and increased bilirubin levels in neonates.

**Figure 4:**
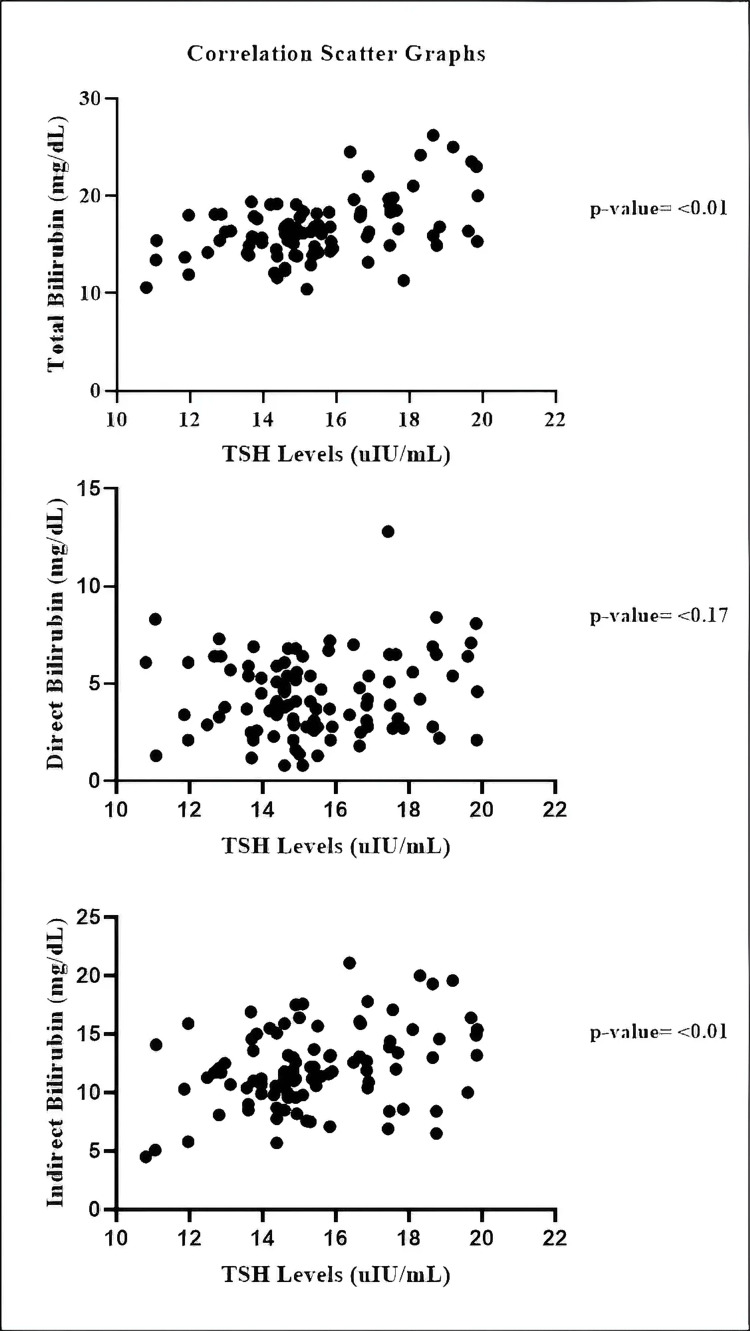
Association of bilirubin and TSH levels in the hypothyroid group (Pearson's correlation) TSH: thyroid-stimulating hormone

A statistically significant positive correlation exists between TSH levels and total bilirubin levels in hypothyroid neonates (r = 0.4577, p < 0.01). Also, a statistically significant positive association exists between TSH levels and indirect bilirubin levels in hypothyroid neonates (r = 0.3255, p < 0.01). However, there was a non-significant correlation observed between TSH levels and direct bilirubin levels (r = 0.1369, p = 0.17) (Figure 4).

## Discussion

The researchers evaluated the bilirubin metabolic relationship with CH through demographic and clinical and biochemical assessments of 100 infant subjects. The research data demonstrates substantial variations between infant patients who suffer from hypothyroidism and those who possess normal thyroid function. Research results provide critical knowledge about CH biochemical behavior, along with evidence for using bilirubin evaluation as a starting indicator of thyroid dysfunction during newborn testing. Postnatal screening needs prompt action because most neonates fall into the 1-6 days age category, where jaundice appears first, since they constitute 40% of the total population. The recorded clinical symptom affected the largest number of neonates with CH through structurally prolonged jaundice (74%) due to its acknowledged position as an initial and frequent indicator of hypothyroidism in newborns [7].

Neonates with CH showed elevated total bilirubin (15.59 ± 2.37 mg/dL) along with direct (4.94 ± 5.27 mg/dL) and indirect bilirubin levels (11.42 ± 3.22 mg/dL) than those observed in the control group where the mean total bilirubin levels were 5.92 ± 2.71 mg/dL and direct bilirubin was 2.63 ± 2.40 mg/dL and indirect bilirubin was 4.32 ± 8. The CH group presented elevated TSH values at a mean level of 15.42 ± 2.08 mIU/L, whereas the control group maintained a mean level of 2.12 ± 0.85 mIU/L. The current research results demonstrated significant statistical significance (p < 0.01), matching the diagnostic standards for CH, by showing elevated TSH levels greater than 10 mIU/L during the newborn period [18].

Data obtained through correlation analysis established the link between thyroid dysfunction and the changed bilirubin metabolic processes. The statistic reveals that TSH values demonstrate a strong positive relation with total bilirubin levels (r = 0.453, p < 0.01) as well as with indirect bilirubin values (r = 0.325, p < 0.01). The weak relationship between TSH levels and direct bilirubin (p = 0.17) indicated that CH patients exhibit increased unconjugated hyperbilirubinemia, mainly due to impaired liver conjugation activities. The results match the research of another article that demonstrated comparable TSH and bilirubin level correlations in hypothyroid neonates [19].

Multiple normal body functions contribute to understanding how hypothyroidism affects the development of hyperbilirubinemia. The hepatic enzyme system, especially uridine diphosphate-glucuronosyltransferase (UGT1A1), responsible for bilirubin conjugation, depends heavily on thyroid hormone thyroxine (T4) for proper regulation. A lack of thyroid hormones delays UGT1A1 enzyme maturation along with its function, which causes bloodstream bilirubin levels to build up [20]. Simultaneously, hypothyroidism weakens gastrointestinal function, which delays meconium clearance and raises bilirubin reabsorption in the gut, resulting in more severe jaundice [21].

Researchers have usually found a solid link between CH and long-lasting neonatal jaundice [22,23], yet select studies point toward a weak connection between thyroid hormone levels and neonatal hyperbilirubinemia [24,25], thus suggesting that jaundice sometimes stems from other thyroid-related conditions. Most of the CH neonates (95%) lacked a family history of CH in this population, thus demonstrating that sporadic cases are common compared to family-based cases, which supports universal screening programs.

Various reports from studies have shown significant associations of CH and prolonged neonatal jaundice. However, in Singh et al.'s matched case-control study of 100 jaundiced and 100 non-jaundiced neonates, mean T3, T4, and TSH levels were similar in both groups, and there was no correlation between the thyroid parameters and level of bilirubin (p > 0.01), and thus, they could not infer any association between iodine deficiency, thyroid dysfunction, and neonatal hyperbilirubinemia [26]. In another study, analysis of 146 term neonates revealed no significant correlation between the levels of TSH and total bilirubin on day 3, thus meaning that thyroid function did not predict the severity of neonatal hyperbilirubinemia [27].

Limitations of this study include its relatively small sample size and the fact that data were collected from only two hospitals in Rawalpindi, which may restrict the generalizability of the findings to broader populations. Certain limitations need acknowledgment despite the solid research evidence. The investigation used only 100 participants while confining their assessment to a few clinical institutions, which reduces the potential breadth of outcome relations. The researchers did not check free T4 or total T4 levels, so they failed to verify the hypothyroidism diagnosis and its severity directly. The observation results could have been affected by uncontrolled confounders because the researchers did not account for maternal iodine status in conjunction with birth weight and delivery mode.

The cross-sectional design also limits the ability to establish a causal relationship between elevated TSH levels and increased bilirubin concentrations. The inconsistency in the reported relationship between CH and neonatal hyperbilirubinemia among studies may be ascribed to several methodological and demographic factors. Moreover, the absence of longitudinal follow-up prevented assessment of post-treatment outcomes or the impact of levothyroxine therapy on bilirubin normalization. Additionally, only TSH levels were evaluated, while the inclusion of other thyroid parameters, such as free T4 and total T4, could have provided a more comprehensive understanding of thyroid function in relation to bilirubin metabolism. Moreover, various potential confounding factors were not systematically evaluated, such as neonatal feeding practices (exclusive breastfeeding versus formula feeding), maternal thyroid status during pregnancy, and the timing of bilirubin measurement concerning postnatal age. Variability in these factors may impact bilirubin metabolism and serum levels, consequently influencing the observed associations.

Future multicenter, longitudinal studies utilizing standardized measurement protocols and formal power estimation are necessary to validate and expand upon these findings.

## Conclusions

This study reveals a substantial correlation between CH and increased total, direct, and indirect bilirubin levels in neonates, with persistent jaundice frequently observed as a clinical manifestation. The identified positive associations between TSH levels and bilirubin concentrations indicate a possible association between thyroid disease and compromised bilirubin metabolism. Nonetheless, due to the study's cross-sectional and observational design, causal inferences cannot be determined. Consequently, these findings should be evaluated with caution and regarded solely as corroborative data that reinforces current knowledge, rather than as a foundation for conclusive therapeutic or policy recommendations. Additional extensive, long-term investigations are necessary to validate these correlations and to ascertain their relevance for neonatal screening and care regimens.

Future research should focus on studying a bigger and more heterogeneous population of subjects through multiple medical facilities. The research design should extend its duration to monitor how neonates develop neurologically when they have CH and severe bilirubin issues. Genetic screening combined with maternal thyroid examination assessment would advance our understanding of thyroid dysfunction in neonates. The creation of inexpensive bedside diagnostic equipment that combines TSH and bilirubin testing runs may expedite early detection, especially for regions with scarce resources.

## Appendices

**Table 4:** Structured assessment questionnaire of the study This questionnaire is the original work of the study authors, Alishba Amin and Ali Husnain

Section	Item Code	Question / Response Options
Section A: Demographic Information	A1	Neonate ID / Code: ______________________
	A2	Age (in days): □ 1–6 □ 7–12 □ 13–18 □ 19–24 □ 25–30
	A3	Sex: □ Male □ Female
	A4	Birth Weight (kg): ______________________
	A5	Gestational Age (weeks): ______________________
	A6	Ethnicity: □ Punjabi □ Pashtun □ Sindhi □ Balochi □ Other: ______________________
Section B: Maternal and Family History	B1	Maternal age at delivery: ______ years
	B2	Maternal thyroid disorder during pregnancy: □ Yes □ No If yes: ______________________
	B3	Maternal medication during pregnancy (anti-thyroid or iodine-related): □ Yes □ No If yes: ______________________
	B4	Family history of thyroid disease: □ Yes □ No
	B5	Family history of neonatal jaundice: □ Yes □ No
Section C: Clinical Characteristics of the Neonate	C1	Age at onset of jaundice (days): ______________________
	C2	Type of jaundice: □ Physiological □ Pathological □ Prolonged (>14 days)
	C3	Activity level: □ Normal □ Lethargic □ Irritable
	C4	Clinical symptoms: □ Prolonged jaundice □ Constipation □ Microcephaly □ Umbilical hernia □ Puffy face □ Lethargy □ Other: ______________________
Section D: Laboratory Investigations	D1	Thyroid Profile: TSH (uIU/mL): ____ ; T4 / Free T4: ____
	D2	Bilirubin Levels: Total: ____ mg/dL ; Direct: ____ mg/dL ; Indirect: ____ mg/dL
	D3	Other Tests: Hemoglobin: ____ g/dL ; Liver function: □ Normal □ Abnormal
Section E: Diagnosis and Management	E1	Thyroid status: □ Congenital Hypothyroidism □ Euthyroid
	E2	Treatment started: □ Yes □ No If yes: ______________________
	E3	Phototherapy required: □ Yes □ No
	E4	Duration of hospital stay (days): ______________________
Research Data Confirmation	F1	Inclusion criteria met: □ Yes □ No
	F2	Exclusion criteria applicable: □ Yes □ No If yes: ______________________
	F3	Consent obtained from parent/guardian: □ Yes □ No
